# *De novo* transcriptomic resources for two sibling species of moths: *Ostrinia nubilalis* and *O. scapulalis*

**DOI:** 10.1186/1756-0500-6-73

**Published:** 2013-02-28

**Authors:** Bernhard Gschloessl, Emmanuelle Beyne, Philippe Audiot, Denis Bourguet, Réjane Streiff

**Affiliations:** 1 Centre de Biologie pour la Gestion des Populations (CBGP) UMR INRA-IRD-CIRAD-Montpellier SupAgro, Campus International de Baillarguet, Montferrier-sur-Lez Cedex, 34988, France; 2 Present address: Institute of Human Genetics, CNRS, Montpellier, 34396, France

**Keywords:** NGS, 454 sequencing, Tanscriptome, Moth, European corn borer, Adzuki bean borer, Sibling species, *Ostrinia nubilalis*, *Ostrinia scapulalis*

## Abstract

**Background:**

This study aimed at enhancing the transcriptomic resources for two sibling species of moths, *Ostrinia scapulalis* (Adzuki bean borer) and *Ostrinia nubilalis* (European corn borer), as a foundation for future researches on their divergence history. Previous works on these species had shown that their genetic divergence was low, while they were reproductively isolated *in natura* and specialized on different host plants. Comparative genomic resources will help facilitate the understanding of the mechanisms involved in this isolation and adaptation to the host plants. Despite their fundamental interest, these species still lack the genomic resources to thoroughly identify candidate genes for functions of interest. We present here a high throughput sequencing and *de novo* transcriptome assembly for these two sibling species in line with this objective of comparative genomics.

**Results:**

Based on 322,504 and 307,622 reads of 454 sequencing for *O. scapulalis* and *O. nubilalis* respectively, we reconstructed 11,231 and 10,773 transcripts, of which 40% were functionally annotated by BLAST analyzes. We determined the level of completeness of both assemblies as well as the recovery level of published *Ostrinia* genomic resources. Gene ontology (GO) of common and species-specific *de novo* transcripts did not reveal GO terms significantly enriched in one or the other species. By applying stringent homology searches on transcripts common to *O. scapulalis* and *O. nubilalis*, we identified a set of homologous transcripts, with a mean nucleotide identity value of 98.1%. In this set, the most divergent transcripts revealed candidate genes involved in developmental, sensorial and pathogen defense processes.

**Conclusions:**

This data greatly increases the genomic resources of *Ostrinia* species and constitute a solid skeleton for future comparative analyzes of expression or diversity, despite we show that the transcriptomes for both species have not been assembled at full completion. In addition, we provide a set of homologous transcripts together with their annotation as a source of candidate genes for comparative analyzes.

## Background

The genus *Ostrinia* comprises about 20 species [1], including two major pests of maize: the European corn borer (ECB, *Ostrinia nubilalis* Hübner) in Europe and USA and the Asian corn borer (ACB, *O. furnacalis* Guéné) in Asia. In sympatry with these two species, and over a large common geographical range, a third species, the Adzuki bean borer (ABB, *O. scapulalis* Walker) feeds on various dicotyledons and spreads over Europe and Asia [2]. In Western Europe, several studies showed that *O. scapulalis* and *O. nubilalis* are sibling species (*i.e.* morphologically indistinguishable) and that their genetic differentiation is rather low (differentiation level, estimated by *F*_st_, ranging from 0.015 to 0.084 on isozyme, microsatellite and Amplified Fragment Length Polymorphism (AFLP) neutral markers [3-5]). In natural populations, these species are reproductively almost fully isolated [6], whereas fertile and viable hybrids can be obtained in the laboratory [7,8]. Ecological studies have shown that these species mainly differ by their specialization to distinct host plants [7,9,10]; the major hosts being maize for *O. nubilalis* and mugwort (*Artemisia vulgaris* L.), hop (*Humulus lupulus* L.) and hemp (*Cannabis sativa* L.) for *O. scapulalis*. They are also distinguished by the composition of the sex pheromone blend emitted by their females in western Europe [11], with a so-called ‘Z’ strain in *O. nubilalis* and a ‘E’ strain in *O. scapulalis*, while both Z and E strains co-occur in each species *e.g.* in the USA for the ECB and in Japan for the ABB [12-15].

The genetic and genomic architecture of the differentiation between *O. scapulalis* and *O. nubilalis* has been estimated based on various low-throughput tools: isozymes [16-19], genes [20], microsatellites [4,21], AFLPs [5]. Because of the restricted number of this type of markers and/or their anonymous location (microsatellites, AFLPs), there has been limited success in precisely identifying the genomic regions or genes implicated in the divergence between *O. nubilalis* and *O. scapulalis*. This divergence is rather low with remarkable exceptions at some markers [5,19,21], suggesting that some regions may carry genes involved in species differentiation and/or in speciation.

Today, we can benefit from next generation sequencing (NGS) technologies to rapidly increase the amount of genomic resources for non-model organisms. In the Sequence Read Archive (SRA) of NCBI (http://www.ncbi.nlm.nih.gov/sra), dedicated to raw sequencing data from the NGS platforms, 482 NGS data sets for 59 Lepidoptera species were available in October 2012, among which 49 correspond to cDNA sequencing from *Bombyx* spp., *Chilo suppressalis, Erynnis propertius, Euphydryas aurinia, Galleria mellonella, Heliconius *spp., *Lymantria dispar, Manduca sexta, Maruca vitrata, Melitaea cinxia, Papilio* spp., *Plutella xylostella, Spodoptera litura, Striacosta albicosta, Zygaena filipendulae*, illustrating the great variety of species and families under current screening.

In the present study, our main challenge was to enhance the genomic resources of *O. scapulalis* and *O. nubilalis* for future comparative researches. We then built a first skeleton of both species transcriptomes from RNA extracted from adult head and thorax tissues sequenced by the 454 technology. We developed an *ad hoc* assembly procedure on more than 300,000 reads for each species and obtained 11,231 and 10,773 transcripts for *O. scapulalis* and *O. nubilalis*, respectively, 40% of which were annotated based on similarities detected by BLAST [22] alignment against the Non-Redundant (NR) NCBI protein database. We also examined the completeness level of both *de novo* transcriptome assemblies based on various statistics of the transcripts, and on recovery estimations of published *Ostrinia* genomic resources. Additionally, we present a first comparative analysis between both transcripts sets based on gene ontology categories and on the sequence identity level of transcripts common to *O. scapulalis* and *O. nubilalis*. These resources, developed in parallel in both *Ostrinia* species, pave the way to researches on their divergent genomic history.

## Results

### Assembly and quality assessment

The original data set from the sequencing center contained 322,504 and 307,622 expressed short sequence reads for *O. scapulalis* and *O. nubilalis*, respectively. Sequence filtering resulted in 287,429 (*O. scapulalis*) and 272,490 (*O. nubilalis*) short reads, respectively. Newbler (version 2.5.3, Roche) assembled 11,231 and 10,773 transcripts (Newbler “isotig”) for *O. scapulalis* and *O. nubilalis*, respectively. Finally, 8,892 unigenes (Newbler term “isogroup”) were identified for *O. scapulalis* and 8,656 for *O. nubilalis*. Table 1 lists the main features to the two transcriptome raw read data and assemblies.

**Table 1:** 454 read and assembly features

	***O. scapulalis***	***O. nubilalis***
Raw short sequence number	322,504	307,622
Number after filtering	287,429	272,490
Mean read length (bp)	352	356
Number of singletons	85,070	87,110
Number of assembled reads	145,588	130,897
Assembled transcriptome (Mbp)	10.35	9.52
Reads/bp	9.8	10.2
Number of unigenes	8,892	8,656
Number of transcripts (including alternative transcripts)	11,231	10,773
Transcript mean length (bp)	922	883
Transcript median length (bp)	693	685
Transcript N50 length (bp)	1,036	991
Mean exon count per transcript	1.54	1.49
Mean exon* length (bp)	604	605
Median exon length (bp)	500	495

* Each contig of an alternative transcript was only counted once as exon.

Newbler predicted 1.26 (*O. scapulalis*) and 1.24 (*O. nubilalis*) alternative transcripts in average *per* unigene, and 1.5 exons (Newbler “contig”) in average *per* transcript (for both species). The mean transcript length was 922 bp (*O. scapulalis*) and 883 bp (*O. nubilalis*), and the mean exon length was 604 bp and 605 bp. “Exons” were here taken as an output of the assembly (*i.e.* when several alternative transcripts composed an isotig, the discrete units of each transcript, the contigs, were considered as exons) and not as a result of a gene structure analysis. The average bp-coverage was 9.8 reads/bp for *O. scapulalis* and 10.2 reads/bp for *O. nubilalis*.

### Low impact of homopolymers on the assembly

It has been shown that 454 sequencing errors in homopolymer regions can hamper a proper DNA assembly [23,24]. We therefore analyzed the lengths of homopolymers in the set of cleaned sequence reads in both sibling species. We first identified the longest homopolymers (at least two subsequent identic bp) for every nucleotide (A, C, G, T). The mean lengths of the longest homopolymers were low for *O. nubilalis* (see Additional file 1: Figure S1; A=2.5 bp, C=2.2 bp, G=2.2 bp, T=2.5 bp) and *O. scapulalis* (see Additional file 2: Figure S2; A=2.5 bp, C=2.2 bp, G=2.2 bp, T=2.5 bp). Similarly, after assembly, the mean lengths of the longest homopolymers in the assembled transcripts were low (see Additional file 3: Figure S3 and Additional file 4: Figure S4; *O. nubilalis*: A=2.4 bp, C=2.2 bp, G=2.2 bp, T=2.4 bp; *O. scapulalis*: A=2.4 bp, C=2.2 bp, G=2.1 bp, T=2.4 bp). Gilles *et al*. [24] recommended for 454 GS-FLX Titanium read assembly a sequencing coverage of at least five reads/bp in order to reduce the presence of sequencing errors such as homopolymers. The assemblies of both *Ostrinia* species fulfilled this criterion with in average about ten reads/bp per transcript. Before the assembly, the homopolymer lengths ranged from 2 to 22 bp in the cleaned reads of both species. After the assembly, the homopolymer lengths of the transcripts ranged between 2 and 11 bp, stating an improvement due to sequence coverage. We identified only one exception, in *O. nubilalis*, where a transcript (identifier “isotig03190”) included a G-homopolymer of 44 bp. This transcript of length 301 bp could not be annotated and had a mean coverage of 6.1 reads/bp which is close to the requested minimum coverage threshold. This indicates that the present homopolymer reads in this specific transcript could not be corrected by the lower coverage.

### Transcript annotation

We assigned molecular functions to the assembled transcripts of each species by screening the NR database for similar protein sequences. We found that 39.6% (4,445 transcripts) of *O. scapulalis* transcripts and 38.4% (4,133 transcripts) of *O. nubilalis* transcripts had hits in the NR database. In addition, 30.1% (25,638 singletons) and 29.5% (25,711 singletons) of the respective *O. scapulalis* and *O. nubilalis* singletons also displayed significant homologies.

Among the best blastx hits, lepidopteran species such as *Danaus plexippus* (*O. scapulalis*: n = 2,619, *O. nubilalis*: n = 2,405), *Bombyx mori* (n = 392 and n = 383), *Manduca sexta* (n = 69 and n = 72) were the most represented (see Additional file 5: Figure S5).

### Assignment of GO-terms

We applied Blast2GO [25] to assign GO-terms to each assembled transcript. GO terms were associated to 2,774 (20.3%) of the *O. scapulalis* transcripts and 2,084 (19.3%) of the *O. nubilalis* transcripts with significant hits in NR database. The distributions of GO terms categories for *O. nubilalis* and *O. scapulalis* were highly similar (see Additional file 6: Figure S6).

### Determination of assembly completeness

We gave a particular attention in this *de novo* transcriptome assembly to the transcripts’ completeness. First, we applied FrameDP [26], an application designed to predict the presence and completeness degree of coding sequences (CDS) in the transcripts. Among the *O. scapulalis* and *O. nubilalis* transcripts, 4,168 (37.1%) and 3,808 (35.4%) were predicted to possess at least partial CDSs, respectively. Of these, complete CDSs were predicted for 1,911 (17%) *O. scapulalis* transcripts and 1,709 (15.9%) *O. nubilalis* transcripts. In the *O. scapulalis* assembly, the CDSs of 1,192 sequences were incomplete at the N-terminus, 594 at the C-terminus and 471 at both termini. For *O. nubilalis*, 1,105 sequences were fragmentary at the N-terminus, 528 at the C-terminus and 466 at both sequence ends (see Table 2).

**Table 2:** FrameDP predictions on transcripts

	***O. scapulalis***	***O. nubilalis***
C-term fragment	594	528
C-term fragment, N-term fragment	471	466
N-term fragment	1,192	1,105
Complete CDS	1,911	1,709
Total predicted	4,168	3,808

A CDS is that part of the exonic region of a gene which is actually coding for a protein (the other exonic regions being 5^′^UTR and 3^′^UTR). The N-terminus is the amino terminus of a protein, and the C-terminus represents the carboxyl terminus.

Then, we extended the estimation of the assembly completeness by calculating the Ortholog Hit Ratios (OHRs) as defined by O'Neil *et al.*[27] (see Methods). The mean OHR were 0.61 (*O. scapulalis*) and 0.62 (*O. nubilalis*). Among all functionally annotated transcripts 2,015 *O. scapulalis* transcripts (45.3%) and 1,969 *O. nubilalis* transcripts (47.6%) had an OHR of at least 0.7 (see Additional file 7: Table S1). Finally, 488 *O. scapulalis* transcripts and 469 *O. nubilalis* transcripts were assembled at full-length (OHR = 1.0) to the respective homologous sequence of the NR database.

### Comparison of *de novo* assemblies with already published *Ostrinia* genomic data

All *Ostrinia* expressed sequence tags (ESTs, n = 14,918) available in the NCBI EST database were downloaded in February 2012. They were then aligned with BLAST against the *de novo* transcriptomes. We found that 32.3% (33.8%) of the ESTs had similarities with the *O. scapulalis* (*O. nubilalis*) *de novo* transcript set. For half of these transcripts, the OHR values were ≥ 0.7 (see Additional file 7: Table S2). When blasting each species-specific transcript set against the NCBI ESTs we identified 8,249 *O. scapulalis* transcripts and 7,958 *O. nubilalis* transcripts that did not have any hit in the *Ostrinia* NCBI ESTs. This indicates that our *de novo* assemblies identified formerly unknown *Ostrinia* transcripts.

In addition, we compared both *de novo* transcript sets to published data issued from independent Sanger and 454 sequencing on specific tissues of *Ostrinia* species [28-30]. Among the 7,413 assembled contigs of that study, 62.3% (n = 4,619) could be aligned to our *O. scapulalis* assembly and 63.3% (n = 4,691) to the set of *O. nubilalis* transcripts with mean OHR values of 0.79 for both species (see Additional file 7: Table S3). *Vice versa*, we determined that 6,293 and 6,024 transcripts were specific to our *de novo O. scapulalis* and *O. nubilalis* assemblies.

In total, in the current study we identified more than 5,500 new transcripts to each of both *Ostrinia* species (n = 5,818 in *O. scapulalis*, n = 5,516 in *O. nubilalis*), which were not contained in the public databases. Of the sets of *de novo* assembled unigenes, 4,841 *O. scapulalis* (comprising 5,526 transcripts) and 4,612 *O. nubilalis* unigenes (comprising 5,253 transcripts) were unique and have not been described at all.

### Divergence between sibling species

8,907 (79.3%) and 8,701 (80.8%) transcripts in *O. scapulalis* and *O. nubilalis* could be aligned to the respective sibling species. Among the similar transcripts a high mean OHR value of 0.72 was observed (see Additional file 7: Table S4).

Overall, 2,324 (20.7%) *O. scapulalis* transcripts and 2,072 (19.2%) *O. nubilalis* transcripts were specific to their respective species assembly. Among these, 1,141 *O. scapulalis* transcripts and 997 *O. nubilalis* transcripts had hits in the NR database with respective mean OHR values of 0.51 and 0.54 (see Additional file 7: Table S5). As one of the main interests of our research on *Ostrinia* is focused on divergent genomic regions linked to the specific host adaptation of each sibling species, we addressed the question whether these private transcripts may constitute a set of Potential Differentially Expressed Genes PDEGs, [31]. The PDEG GO term distributions were indeed highly similar between *O. scapulalis* and *O. nubilalis.* We tested with Fisher’s exact tests in Blast2GO if the GO term associations to each species PDEG set were significantly different from the set of all GO terms to the same species. These enrichment analyzes revealed no GO terms being significantly enriched in the PDEGs of *O. scapulalis* and *O. nubilalis* (*i.e.* p > 0.05 after correction for multiple testing, see Additional file 7: Table S6).

**Figure 1:**
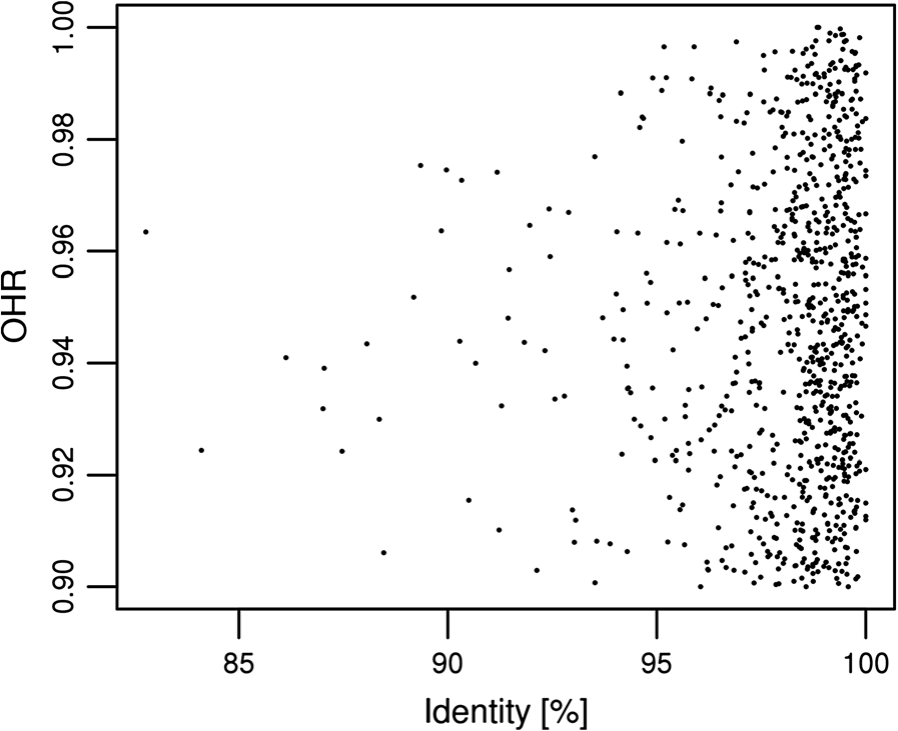
**Distribution of identity *versus *OHR of homologous transcripts.** Identities *versus* OHRs are given for homologous transcripts between *O. scapulalis* and *O. nubilalis* based on the best reciprocal hit.

In order to analyze the divergence among the orthologous genes of both sibling species we determined their level of identity. Homologous transcripts between both species were determined by limiting the set of common transcripts to those with the corresponding reciprocal best hit (RBH) determined with stringent criteria (*i.e.* e-value ≤ 1e-10, sequence overlap ≥ 90%). This conservative procedure aimed at avoiding false positives. These conditions were fulfilled for 846 transcripts. Among them the identity percentage varied between 82.8% and 100% (mean identity = 98.1%) (see Figure 1), with half of the transcripts showing a high sequence identity of at least 98.7%. By contrast, 146 homologs had a slightly lower identity (between 82.8 and 97%). Focusing on these most divergent homologs, we could assign 41 functional *via* BLAST similarity searches against NR (see Additional file 7: Table S7). About half of these were annotated as “hypothetical proteins”. Among the others were genes involved in regular cell processes and apoptosis (ribosomal protein, NADH, reverse transcriptase, ubiquitins), in development and moulting (myophilin, cuticlin, cathepsin, ADAMT), in sensory traits (olfaction with GTP protein, gustatory receptor, and vision with myosin III) and in defense against pathogens (antiviral response with serpine2, immediate early response interacting protein putatively involved in response to pathogens, Hdd1 protein putatively involved in immune regulation against bacteria infestation).

## Discussion

Lepidoptera constitute the second major group of insects [32] and a major source of agricultural pests, which justifies the need of increasing genomic resources for numerous species of this group. Indeed, at the time of this study, only the genomes of four lepidopteran species are completely sequenced and accessible through biological databases (*Bombyx mori*[33], *Heliconius melpomene*, *Danaus plexippus* and *Plutella xylostella*, http://www.ncbi.nlm.nih.gov/bioproject/71053,72423,73593,73595,73597,73599, PRJNA78271). The decreasing costs of NGS provide a higher accessibility to small research groups. Hence, the number of lepidopteran species transcriptomes and genomes sequenced by NGS [34-37] is increasing. The present study aimed at reconstructing the set of expressed genes of two moth species, one of which being a major pest of maize. Previous studies identified the ecological, behavior and physiological differences between these species, while very little is known on the genetic architecture underlying these traits [but see [38], on pheromonal pathways]. Our *de novo* transcriptomes offer a major contribution on deciphering the expressed sequences, with approximately 9,000 reconstructed unigenes for each sibling-species, with more than 4,600 unigenes newly identified for each species. The number of predicted genes for well-established insect genomes such as *Bombyx morii* genome size 530 Mbp: 14,623 genes [33], *Drosophila melanogaster* 165 Mbp: 14,039 genes [33] and *Anopheles gambiae* 278 Mbp: 13,683 genes [39] suggests that we did not reach the completion of the transcriptome for each species. This is also apparent through the assembly features, OHR values (annotated transcripts cover about 60% of the total length of the hits by BLAST alignment against the NR database) and CDS predictions (only 20% of the transcripts contain a complete CDS).

One possible explanation for the results is the relatively low sequencing depth. *De novo* NGS transcriptome sequencing data of non-model lepidopteran species provide insights into the relation between the necessary sequencing effort when using 454 technology and the obtained transcriptome completeness: 3,500 contigs for 88,841 reads in *Maruca vitrata*[35], 30,000 contigs in *Zygaena filipendulae* for 319,000 reads [40], 48,354 contigs for more than 600,000 reads in *Melitaea cinxia*[41], 18,000 contigs for 789,105 reads in *Galleria mellonella*[36], and 19,000 contigs for 1.8 M reads in *Manduca sexta*[37]. *Ca.* 400,000 reads led to an assembly of 18-20 Mbp in *Erynnis propertius* and *Papilio zelicaon*[27]. The relationship between the number of reads and the number of contigs is thus positively correlated until a given threshold, at which the number of contigs reaches a plateau probably converging towards the real number of transcripts, while increasing the sequencing depth. Ewen-Campen *et al.*[42] propose at least 1.5 million short read sequences when using 454 NGS technology in order to cover all transcripts. More than the transcript amount and length, this effort also increases the sequencing depth (*e.g.* 23.2 reads/bp for their study and about 10 reads/bp in the present data).

Besides the sequencing depth effect, the single stage sampling (adult) certainly explains the reason we did not detect transcripts specific from other stages. Actually, we decided to focus on the adult stage, and on the thorax and head organs, because key genes are known to express at this stage. These key genes are for instance involved in the recognition of sex pheromonal blends by males [11], in the *Am* trait for assortative mating [8] acting as a short range reproductive barrier between adult males and females of *O. scapulalis* and *O. nubilalis*, and in the host preference for oviposition by gravid females [7,10]. Little is known in insects about the genetic architecture underlying this latter trait, but olfactory genes appear to be good candidates [43]. Our *de novo* transcriptome largely recovered the transcripts assembled by Wanner *et al*. [30] after RNA sequencing expressed in *Ostrinia* antenna. These transcripts specifically target olfaction genes and our new assemblies provide thus additional data to compare or define specific single nucleotide polymorphisms (SNP). The choice of the adult stage and specific organs in the present study resulted from a compromise between transcripts coverage in number and sequencing depth. More sequencing will be necessary to target other organ- or stage- specific transcripts.

Keeping in mind the coverage limitations in the present study, we performed a rough comparative analysis of the *O. nubilalis versus O. scapulalis* assemblies. More generally, we explored the common *versus* species-specific transcripts observed in both *de novo* assemblies. No molecular function appeared to be over- (under-) expressed in either one or the other species (as shown by GO term analysis). We may attribute this result in part to the normalization process of the transcripts before sequencing. This normalization aimed at recovering the lowest expressed transcripts and reducing the most expressed ones. *De facto*, the variations in expression of the RNA transcripts were eliminated. Yet, this process is never 100%-efficient and other studies could detect PDEGs even after normalization [31], especially when comparing two species with a higher expected divergence than populations or stages within a species. Based on the assembly features and results of the annotation analyzes we assume that the sequencing coverage in the present study was insufficient to circumvent the normalization effect.

At the sequence level, we observed a high-level of identity in average between both *Ostrinia* species (mean identity = 98.1%). In order to avoid false positives while searching for homologs between species, we applied stringent criteria (in overlap and e-value) that may have slightly biased these values towards higher identities. However, the observations of the present study at the transcriptomic level are congruent with previous identity estimations at the genomic level between *O. nubilalis* and *O. scapulalis*[4,5]. Since our general interest concerns all divergence events between these *Ostrina* species, at the expression level (PDEG) and as well at the sequence level, we particularly focused on the 146 most diverging genes (*i.e.* in an 82-97% identity range). Due to the limited current resources in public sequence databases most of these genes could not be functionally annotated. Among those that could be annotated were functions involved in development, defense against pathogens and sensory processes. These life history traits are good candidates in the divergence process among phytophagous insects in general [44], and in *Ostrinia* in particular [7,19]. No data were previously available in *Ostrinia* for the *genes* themselves involved in such traits. While validation studies are essential to resolve their pertinence in the divergence process between *Ostrinia* spp., the present gene set enlarges the pool of available candidate genes.

## Conclusions

Altogether, the data we present in this study represent a significant gain in genomic resources for these two moth species *per se* and will contribute to future comparative analyzes and to a better comprehension of their common history. In particular, these new resources represent a great potential for molecular marker discovery (SNPs and microsatellites) dedicated to population analysis, especially since microsatellites are complex to develop, limited in Lepidoptera in general ([45] but see [46]), and to date limited to a little dozen in *Ostrinia*[47,48]. Gene-centered SNPs offer a great potential in genome scan analyzes [49] as they remove the anonymity we were confronted to with AFLP markers for example [5]. The average coverage (*ca*. 10 reads/bp) reached here for each sibling species allows SNPs discovery with the restriction that all positions with lower coverage will likely generate a high rate of false SNPs (see table three in [50]). This argument and former ones (transcripts number, length, CDS, and OHR) encourage us to pursue the sequencing effort for these two sibling species by mapping short reads on these *de novo* skeletons to give confidence to mutation discovery both at the intra- and inter-species level.

## Methods

### Preparation of two random-primed/normalized cDNA libraries from *Ostrinia* spp. for GS FLX Titanium sequencing

We commissioned two random-primed normalized cDNA libraries for *O. nubilalis* and *O. scapulalis* species by a third-party service provider (GATC Biotech, Konstanz, Germany). Briefly, in 2009, moth larvae were collected in natural populations of northern France (Loos en Gohelle) in maize stacks and mugwort. *Ostrinia* larvae were raised to the adult stage in our laboratory. No diagnostic marker, neither a morphological nor a molecular one, is to date available to identify the species at the individual level. Yet, previous studies showed that in northern France, a clear assignation to host-affiliated species (*i.e*. *O. nubilalis* on maize and *O. scapulalis* on mugwort) was observed based on multilocus clustering analyzes, and that the hybridization level was highly restricted [4-6]. Hence, we refer hereafter to *O. scapulalis* for samples collected on mugwort and to *O. nubilalis* for those collected on maize, while the presence of hybrids in the sample is poorly supported. Two pools of heads and thorax of four adult moths (two males and two females) were prepared, one for each species. Samples were ground under liquid nitrogen and the total RNA was isolated from the tissue powder using the mirVana miRNA isolation kit (Ambion). The total RNA preparations were analyzed for their integrity by denaturing agarose gel. From the total RNA, poly(A)+ RNA was prepared. First-strand cDNA synthesis was primed with a N6 randomized primer. Then, the 454 adapters A (CCATCTCATCCCTGCGTGTCTCCGACTCAG) and B (CTGAGACTGCCAAGGCACACAGGGGATAGG) were ligated to the 5^′^ and 3^′^ ends of the cDNA respectively. The cDNAs were finally amplified with PCR (16 cycles for *O. nubilalis* and 15 for *O. scapulalis*), using a proof reading enzyme. Normalization was carried out by one cycle of denaturation and reassociation of the cDNA, resulting in N1-cDNA. Reassociated ds-cDNA was separated from the remaining ss-cDNA (normalized cDNA) by running the mixture over a hydroxylapatite column. After hydroxylapatite chromatography, the ss-cDNA was amplified with 13 PCR cycles. For Titanium sequencing, the cDNA within the range of 500-700 bp were eluted from a preparative agarose gel. Aliquots of the size fractionated cDNAs were analyzed on a 1.5% agarose gel and pooled at equimolar concentrations. The normalized cDNA libraries were sequenced in one GS FLX (Roche 454) standard chemistry run (half a plate for the pool of both species). The cDNAs were double-stranded and composed of the 5^′^-454 adapter, a 6 bp barcode for the *Ostrinia* species identification (ATCAGC for *O. nubilalis* and CACACG for *O. scapulalis*), a 5^′^ adapter (GACCTTGGCTGTCACTC), the EST sequence, a 3^′^ adapter (TCGCAGTGAGTGACAGGCCA) and the 454-3^′^ adapter.

### Sequence cleaning

The 454 adapter sequences located at the termini of the short reads were removed using the sffinfo application of Roche. Subsequently, we ran an in-house pipeline which applies Cross_match (Phrap/Cross_match/Swat package, http://www.phrap.org), SeqClean (http://sourceforge.net/projects/seqclean) and TrimSeq (EMBOSS package version 6.3.1, [51]) in order to clip those adapter sequences which were not correctly removed by the sffinfo program. In addition, this pipeline performed further filtering steps which removed species barcodes, too short sequence reads, sequences with a high ratio of undetermined nucleotides (N) and ribosomic RNA sequences representing eventual bacterial contaminations.

### Assembly of 454 short reads

The short reads were assembled with Newbler (version 2.5.3, Roche). Newbler has the advantageous option, in comparison to other assembly algorithms, to reconstitute alternative transcripts (designed as “isotigs” in Newbler outputs). A Newbler isotig represents a specific transcript composed of successive contigs connected by bridging sequence reads. This specific sequence of contigs is also called contig graph. A Newbler contig itself represents an exon. Alternative transcripts or splice variants are isotigs in which specific contigs are excluded (corresponding to cassette exons) from the contig graph. A Newbler “isogroup” comprises a set of all alternative transcripts that share the same contig subset. In consequence, an isogroup should represent, in theory, a unigene.

We used the following parameters for the design of alternative transcripts: a maximum of 500 contigs *per* isogroup (“-ig” option), a minimum contig length of 100 bp (“-icl” option) and a maximum of 100 isotigs *per* isogroup (“-it” option). Further parameters applied were “-cdna” for transcriptome assembly, “-notrim” to disable additional quality trimming (since cleaning was already established) and “-het” to take into account the high variability in the short sequence reads due to heterozygosity within each of the two lepidopteran species. Regarding clustering, we requested a minimum overlap criterion of 40 bp (“-ml” option) and 97% identity (“-mi” option) for each species.

### Functional annotation of assembled transcripts

For each sibling species assembly, all transcripts were automatically annotated by blastx against the NR database (build of 06/12/2011), applying a threshold e-value of 1e-5. The same filter criterion was applied for the BLAST alignments of the *O. nubilalis* contigs of the assembly of Coates *et al.* ([28], see below for this latter data set). For all blast analyzes we applied a filter on the results in order to keep only the best hits to a specific transcript. More precisely, for each transcript we only retained hits of the best alignment quality defined by a combination of the sequence overlap and the sequence identity. High sequence overlap and identity among the aligned bp (at least 70% overlap and 50% identity) represented the best category, followed by high overlap (≥ 70%) and low identity (< 50%) (second category), low overlap (< 70%) but high identity (≥ 50%) (third category) and low overlap (< 70%) and low identity (< 50%) as fourth and lowest hit category. Only those hits of the respective best-hit category were kept for each transcript for further functional analyzes such as determination of GO terms with Blast2GO and calculation of PDEGs and OHRs. For assignment of GO terms we applied a local installation of the Blast2GO pipeline b2g4pipe (version 2.3.5), using the default options, with access to a local Blast2GO MySQL database (version of june 2011, b2g_jun11). GO term enrichment analyzes were done by running the web application of Blast2GO (http://www.blast2go.com/webstart/makeJnlp.php) on the calculated GO terms.

### Determination of assembly completeness

CDSs were predicted with the FrameDP (version 1.2.0) application [26], applying the default parameters. This way, we allowed similarity searches against well-established Swiss-Prot proteins and self-training in order to optimize the predictions.

As suggested by O'Neil *et al.*[27], we calculated an OHR for each assembled transcript with at least one hit by dividing the length of the ungapped alignment by the total length of the hit sequence. In the current analysis, an OHR (value between 0 and 1) indicates how well a transcript reconstitutes a hit sequence in a known reference set (*e.g.* NR database or known *Ostrinia* gene sets). An OHR of 1 indicates that the assembled transcript covers the entire hit sequence. For transcripts with more than one hit sequence, the hit with the best OHR value was kept as reference for further OHR interpretation.

### Calculation of sequence identity

Transcripts common to both *Ostrinia* species were determined by bi-directional inter-species blastn alignment. As these species are closely related and poorly divergent, we applied stringent criteria to determine the true positive homologs: reciprocal best hits were determined with an e-value of at least 1e-10 and a minimum alignment sequence overlap of 90%. The sequence identity percentage was retrieved from the blastn alignment output features.

### Additional *Ostrinia* data

Public available *Ostrinia* ESTs were downloaded from the NCBI EST (http://www.ncbi.nlm.nih.gov/nucest) by searching for “Ostrinia”[Organism] NOT ribosom* [All Fields]. This search held 14,918 ESTs. The published *O. nubilalis* contig set we used for comparative analysis represented a combination of Sanger sequencing of RNA extracted from larval midgut [28,29] and 454 NGS of RNA extracted from antenna of young adults [30]. After an in-house cleaning procedure, Coates *et al.*[28] used Newbler for the assembly with default parameters, *i.e.* a minimum overlap-length of 40 bp and a sequence identity of 90%. Their resulting set of 7,413 non-redundant contigs displayed a mean contig length of 360 bp (n50 = 392 bp, [157-2,886 bp]) and a total size of the assembly of 2.7 Mbp.

### Availability of supporting data

The raw short read sequences and the assembled transcripts were deposited within NCBI BioProjects (*O. scapulalis*: PRJNA192419 and *O. nubilalis*: PRJNA190899).

## Abbreviations

GO: Gene ontology; ECB: European corn borer; ABB: Adzuki bean borer; AFLP: Amplified fragment length polymorphism; NGS: Next generation sequencing; SRA: Sequence read archive; cDNA: Complementary DNA; NR: Non-redundant NCBI protein database; CDS: Coding sequence; N-terminus: Amino terminus of a protein sequence; C-terminus: Carboxyl terminus of a protein sequence; OHR: Ortholog hit ratio; EST: Expressed sequence tag; PDEG: Potential differentially expressed gene; RBH: Reciprocal best hit; SNP: Single nucleotide polymorphism; ds-cDNA: Double-stranded complementary DNA; ss-cDNA: Single-stranded complementary DNA

## Competing interests

The authors declare that they have no competing interests.

## Authors’ contributions

RS and DB designed the project. BG designed and conducted the bioinformatics analyzes with the support of EB. RS, DB, PA performed the field sampling, and PA the rearing of the samples until the RNA extract. BG, RS, DB and EB wrote the manuscript. All authors read and approved the manuscript.

## Supplementary Material

Additional file 1: Figure S1 Homopolymer length distributions in *O. nubilalis* reads. The distributions of the longest homopolymer in the *O. nubilalis* reads are given. For each transcript the longest homopolymer had to be composed of at least two successive identic nucleotides. The X axis lists the homopolymer length and the Y axis shows the counts of transcripts with homopolymers of the specific length. The figure shows the homopolymer distributions for the nucleotides A, C, G and T, respectively. Click here for file

Additional file 2: Figure S2 Homopolymer length distributions in *O. scapulalis* reads. The distributions of the longest homopolymer in the *O. scapulalis* reads are given. For each transcript the longest homopolymer had to be composed of at least two successive identic nucleotides. The X axis lists the homopolymer length and the Y axis shows the counts of transcripts with homopolymers of the specific length. The figure shows the homopolymer distributions for the nucleotides A, C, G and T, respectively. Click here for file

Additional file 3: Figure S3 Homopolymer length distributions in *O. nubilalis* transcripts. The distributions of the longest homopolymer in the *O. nubilalis* transcripts are given. For each transcript the longest homopolymer had to be composed of at least two successive identic nucleotides. The X axis lists the homopolymer length and the Y axis shows the counts of transcripts with homopolymers of the specific length. The figure shows the homopolymer distributions for the nucleotides A, C, G and T, respectively. Click here for file

Additional file 4: Figure S4 Homopolymer length distributions in *O. scapulalis* transcripts. The distributions of the longest homopolymer in the *O. scapulalis* transcripts are given. For each transcript the longest homopolymer had to be composed of at least two successive identic nucleotides. The X axis lists the hompolymer length and the Y axis shows the counts of transcripts with homopolymers of the specific length. The figure shows the homopolymer distributions for the nucleotides A, C, G and T, respectively. Click here for file

Additional file 5: Figure S5 Species distribution of BLASTs *versus* NR. The most represented species resulting from blastx analyzes *versus* NR (e-value cutoff = 1e-5) are shown for both *de novo* transcriptomes. Click here for file

Additional file 6: Figure S6 GO term distribution. Standard configuration of the Blast2GO web application (http://www.blast2go.com) was applied to generate level 3 graphs for GO-term distributions to the categories molecular function **(Figure S6A)**, biological process **(Figure S6B)**, and cellular component **(Figure S6C)**. No significant differences were observed between the *Ostrinia* GO-terms distributions (Kolmogorov-Smirnov test, P = 0.661, 0.581 and 0.979 for **Figures S6A**, **S6B** and **S6C**, respectively). Abbreviations: NTP: nucleoside-triphosphatase, transmem.: transmembrane, substr.-spec.: substrate-specific **(Figure S6A)**. cell. macromol.: cellular macromolecular **(Figure S6B)**. mem.: membrane **(Figure S6C)**. Click here for file

Additional file 7: Table S1 Ortholog Hit Ratios (OHR) for best hits in NR. **Table S2** Recovery level of NR *Ostrinia* ESTs. **Table S3** Recovery level of a reference and public dataset of *O. nubilalis* contigs. **Table S4** Distribution of Ortholog Hit Ratios (OHR) of similar transcripts. **Table S5** Distribution of Ortholog Hit Ratios (OHR) of PDEGs**. Table S6** Blast2GO Fisher enrichment test results for PDEGs versus complete transcripts set GO terms. **Table S7** NR-Annotation of homologous transcripts between *O. nubilalis* and *O. scapulalis*, with sequence identities below 97%. Click here for file
