# Supplementary material for: De novo transcriptomic resources for two sibling species of moths: Ostrinia nubilalis and O. scapulalis
Source: BMC Res Notes. 2013 Feb 28;6:73. doi: 10.1186/1756-0500-6-73 (PMC3599821; doi:10.1186/1756-0500-6-73)
Supplement: Additional file 1: Figure S1 — Homopolymer length distributions in O. nubilalis reads. The distributions of the longest homopolymer in the O. nubilalis reads are given. For each transcript the longest homopolymer had to be composed of at least two successive identic nucleotides. The X axis lists the homopolymer length and the Y axis shows the counts of transcripts with homopolymers of the specific length. The figure shows the homopolymer distributions for the nucleotides A, C, G and T, respectively. [file 1756-0500-6-73-S1.pdf]

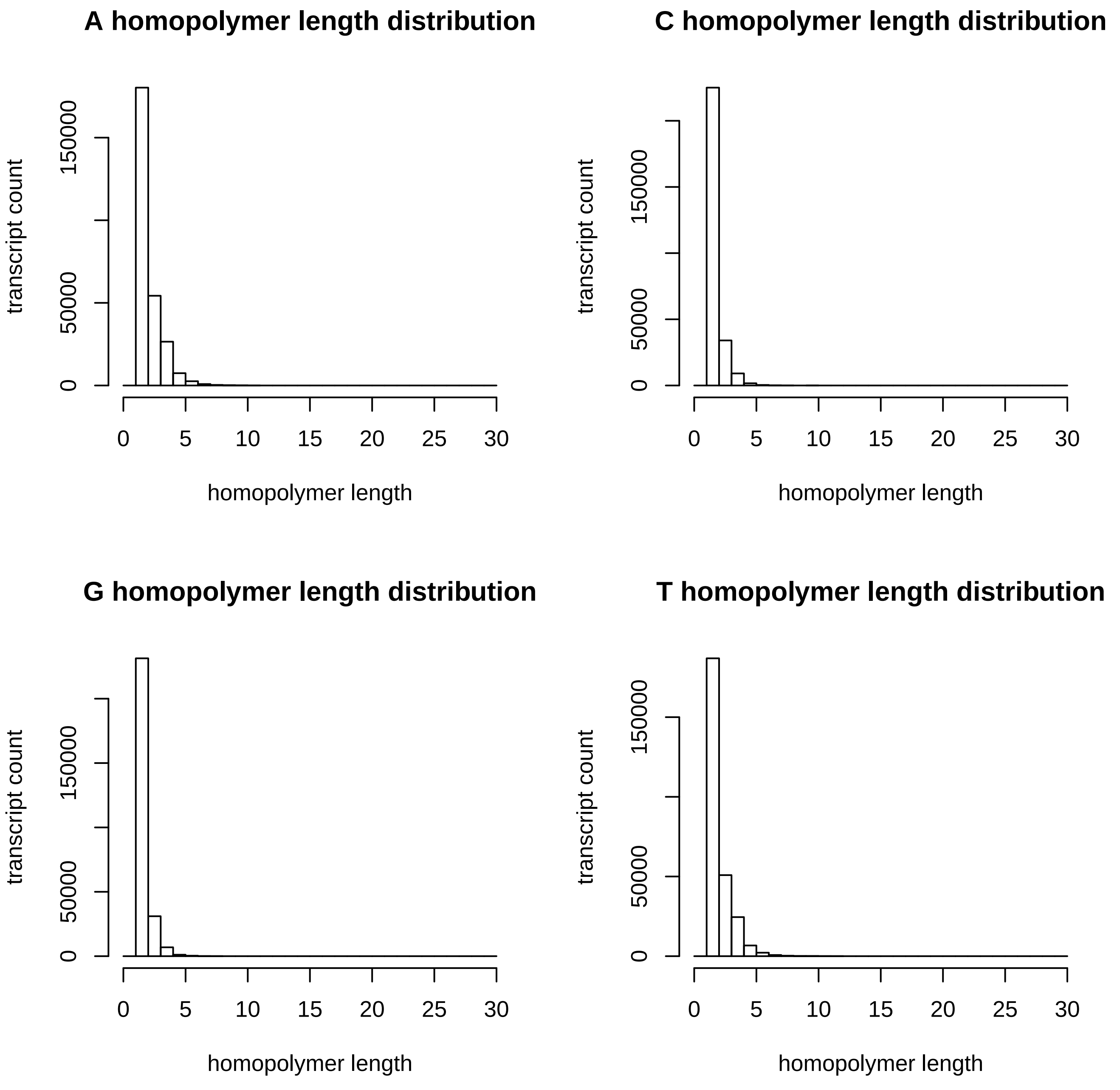
A homopolymer length distribution
C homopolymer length distribution
G homopolymer length distribution
T homopolymer length distribution
transcript count
homopolymer length
0
50000
150000
0
5
10
15
20
25
30
