# Supplementary material for: De novo transcriptomic resources for two sibling species of moths: Ostrinia nubilalis and O. scapulalis
Source: BMC Res Notes. 2013 Feb 28;6:73. doi: 10.1186/1756-0500-6-73 (PMC3599821; doi:10.1186/1756-0500-6-73)
Supplement: Additional file 2: Figure S2 — Homopolymer length distributions in O. scapulalis reads. The distributions of the longest homopolymer in the O. scapulalis reads are given. For each transcript the longest homopolymer had to be composed of at least two successive identic nucleotides. The X axis lists the homopolymer length and the Y axis shows the counts of transcripts with homopolymers of the specific length. The figure shows the homopolymer distributions for the nucleotides A, C, G and T, respectively. [file 1756-0500-6-73-S2.pdf]

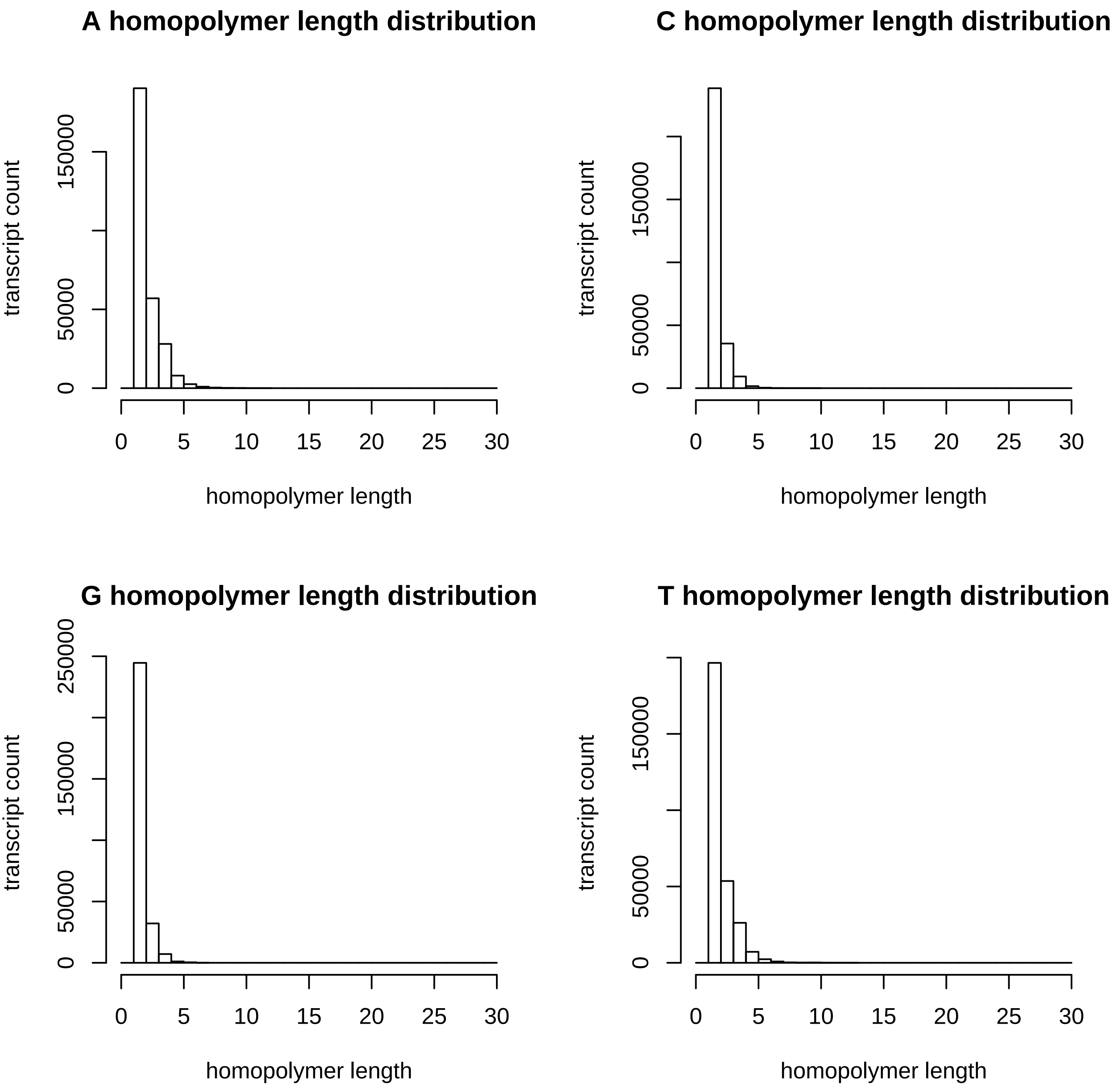

A homopolymer length distribution
transcript count
0
50000
150000
0
5
10
15
20
25
30
homopolymer length
C homopolymer length distribution
transcript count
0
50000
150000
0
5
10
15
20
25
30
homopolymer length
G homopolymer length distribution
transcript count
0
50000
150000
250000
0
5
10
15
20
25
30
homopolymer length
T homopolymer length distribution
transcript count
0
50000
150000
0
5
10
15
20
25
30
homopolymer length
