# Supplementary figures and images for: De novo transcriptomic resources for two sibling species of moths: Ostrinia nubilalis and O. scapulalis
Source: BMC Res Notes. 2013 Feb 28;6:73. doi: 10.1186/1756-0500-6-73 (PMC3599821; doi:10.1186/1756-0500-6-73)

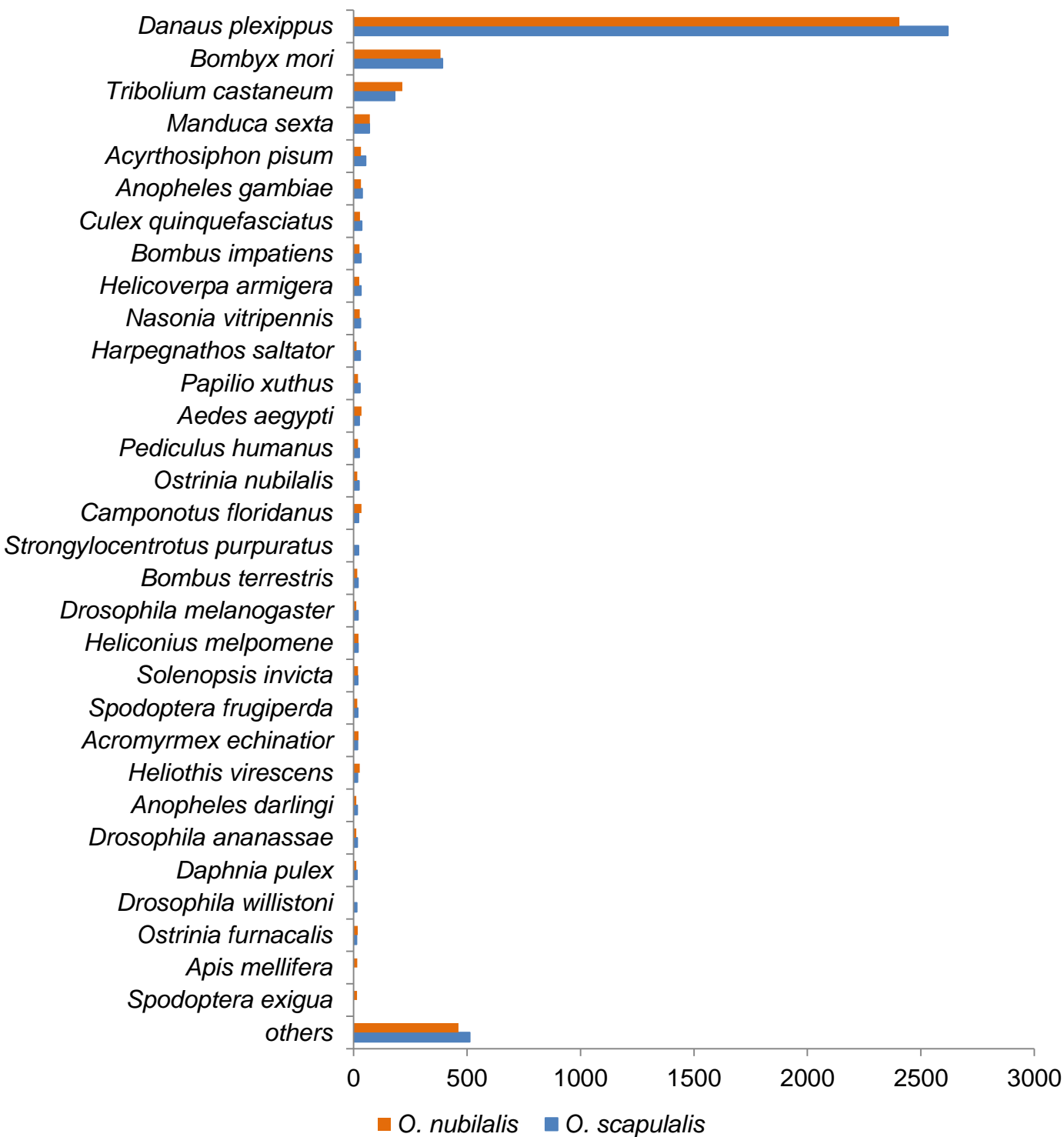

Supplement: Additional file 5: Figure S5 — Species distribution of BLASTs versus NR. The most represented species resulting from blastx analyzes versus NR (e-value cutoff = 1e-5) are shown for both de novo transcriptomes. [file 1756-0500-6-73-S5.pdf]

A

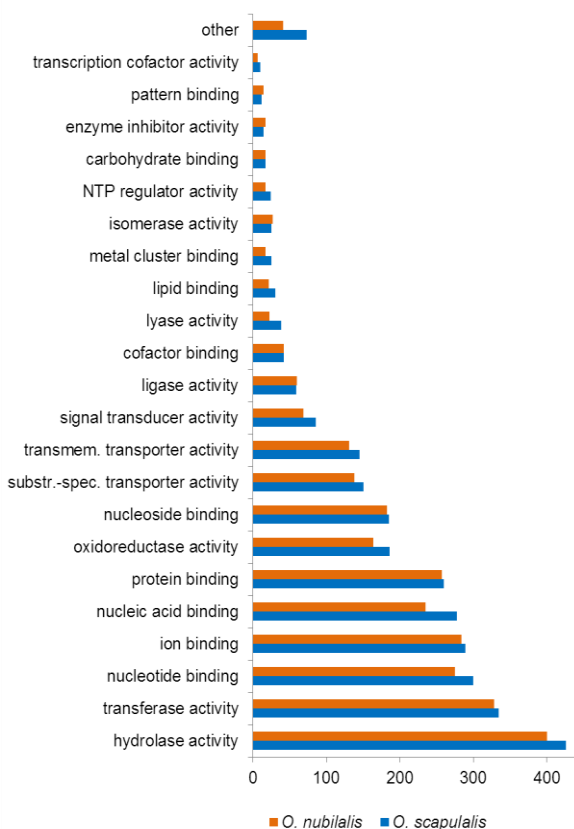

B

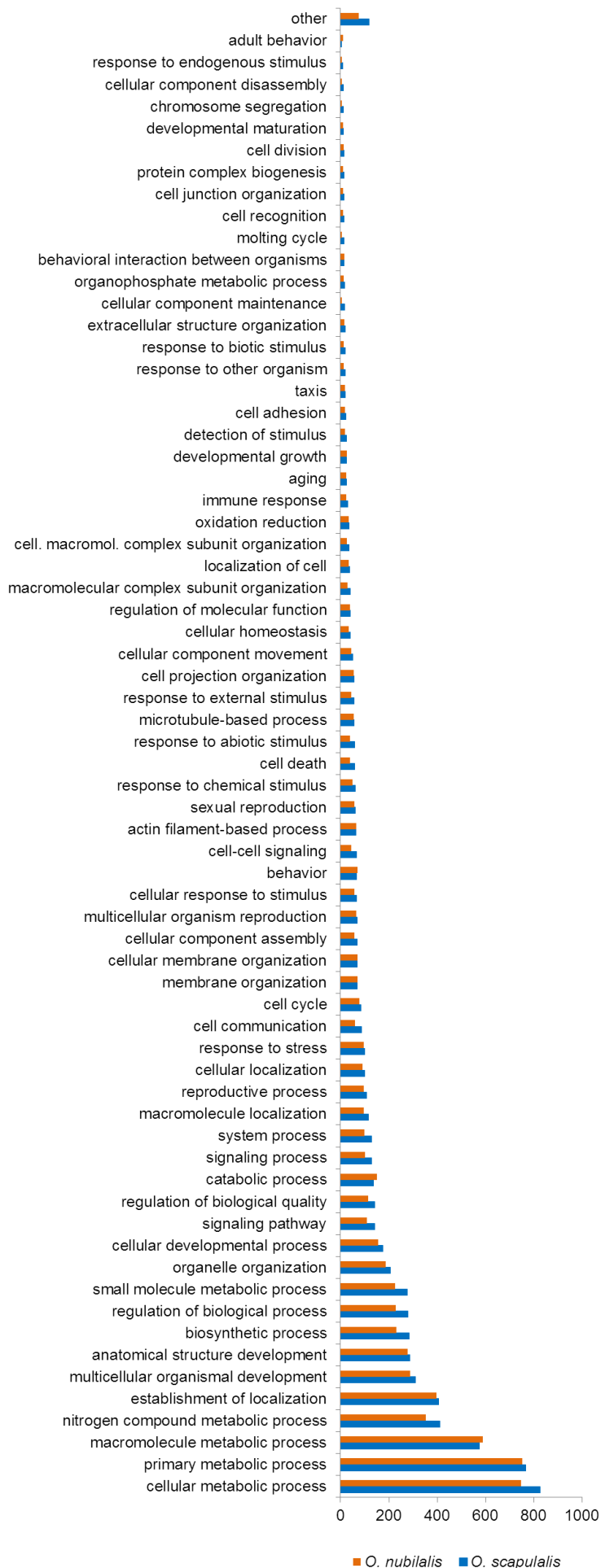

C

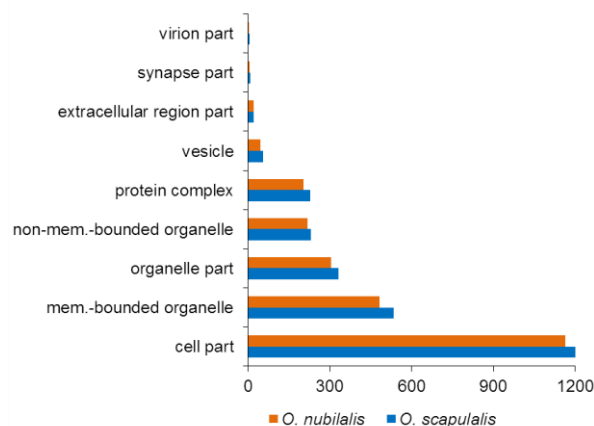

Supplement: Additional file 6: Figure S6 — GO term distribution. Standard configuration of the Blast2GO web application (http://www.blast2go.com) was applied to generate level 3 graphs for GO-term distributions to the categories molecular function (Figure S6A), biological process (Figure S6B), and cellular component (Figure S6C). No significant differences were observed between the Ostrinia GO-terms distributions (Kolmogorov-Smirnov test, P = 0.661, 0.581 and 0.979 for Figures S6A, S6B and S6C, respectively). Abbreviations: NTP: nucleoside-triphosphatase, transmem.: transmembrane, substr.-spec.: substrate-specific (Figure S6A). cell. macromol.: cellular macromolecular (Figure S6B). mem.: membrane (Figure S6C). [file 1756-0500-6-73-S6.pdf]
