# Supplementary material for: De novo transcriptomic resources for two sibling species of moths: Ostrinia nubilalis and O. scapulalis
Source: BMC Res Notes. 2013 Feb 28;6:73. doi: 10.1186/1756-0500-6-73 (PMC3599821; doi:10.1186/1756-0500-6-73)
Supplement: Additional file 7: Table S1 — Ortholog Hit Ratios (OHR) for best hits in NR. Table S2 Recovery level of NR Ostrinia ESTs. Table S3 Recovery level of a reference and public dataset of O. nubilalis contigs. Table S4 Distribution of Ortholog Hit Ratios (OHR) of similar transcripts. Table S5 Distribution of Ortholog Hit Ratios (OHR) of PDEGs. Table S6 Blast2GO Fisher enrichment test results for PDEGs versus complete transcripts set GO terms. Table S7 NR-Annotation of homologous transcripts between O. nubilalis and O. scapulalis, with sequence identities below 97%. [file 1756-0500-6-73-S7.doc]

| OHR | *O. scapulalis* | *O. nubilalis* |
| --- | --- | --- |
| [0.0-0.1]  [0.1-0.2]  [0.2-0.3]  [0.3-0.4]  [0.4-0.5]  [0.5-0.6]  [0.6-0.7]  [0.7-0.8]  [0.8-0.9]  [0.9-1.0] | 301  434  324  306  343  317  405  379  415  1221 | 241  383  338  287  272  315  328  346  438  1185 |

**Table S1 : Ortholog Hit Ratios (OHR) for best hits in NR.**
The table lists the reconstitution level (OHR) of the best hit in the NR database for all annotated transcripts of each sibling-species assembly.

| OHR | min. | 1^st^ quartile | median | mean | 3^rd^ quartile | Max. |
| --- | --- | --- | --- | --- | --- | --- |
| *O.scapulalis* | 0.04 | 0.32 | 0.74 | 0.63 | 0.96 | 1.00 |
| *O.nubilalis* | 0.04 | 0.30 | 0.69 | 0.62 | 0.93 | 1.00 |

**Table S2 : Recovery level of NR *Ostrinia* ESTs.**The table lists the Ortholog Hit Ratios (OHR) of *Ostrinia* ESTs obtained from the NCBI EST database for each *de novo* assembly.

| OHR | *O. scapulalis* | *O. nubilalis* |
| --- | --- | --- |
| [0.0-0.1]  [0.1-0.2]  [0.2-0.3]  [0.3-0.4]  [0.4-0.5]  [0.5-0.6]  [0.6-0.7]  [0.7-0.8]  [0.8-0.9]  [0.9-1.0]  total | 23  145  182  189  234  267  310  380  642  2247  4619 | 15  148  180  198  239  276  302  394  637  2302  4691 |

**Table S3: Recovery level of a reference and public dataset of *O. nubilalis* contigs.**

The OHRs for transcripts with homologous sequences of an independent *O. nubilalis* sequencing and *de novo* assembly are listed for each species assembly of this study.

1. Coates BS, Bayles DO, Wanner KW, Robertson HM, Hellmich RL, et al. (2011) The application and performance of single nucleotide polymorphism markers for population genetic analyses of Lepidoptera. Frontiers in genomic assay technology 2:38.

2. Coates BS, Sumerford DV, Hellmich RL, Lewis LC (2008) Mining an *Ostrinia nubilalis* midgut expressed sequence tag (EST) library for candidate genes and single nucleotide polymorphisms (SNPs). Insect Molecular Biology 17: 607-620.

3. Wanner KW, Nichols AS, Allen JE, Bunger PL, Garczynski SF, et al. (2010) Sex pheromone receptor specificity in the European corn borer moth, *Ostrinia nubilalis*. PLoS ONE 5(1): e8685.

| OHR | *O. scapulalis*  *vs*  *O. nubilalis* | *O. nubilalis*  *vs*  *0. scapulalis* |
| --- | --- | --- |
| [0.0-0.1]  [0.1-0.2]  [0.2-0.3]  [0.3-0.4]  [0.4-0.5]  [0.5-0.6]  [0.6-0.7]  [0.7-0.8]  [0.8-0.9]  [0.9-1.0]  total | 157  323  388  499  662  848  862  841  984  3343  8907 | 166  312  332  429  584  803  742  807  988  3538  8701 |

**Table S4: Distribution of Ortholog Hit Ratios (OHR) of similar transcripts.**Table lists the distribution of OHR values of a transcript of one species aligned with the ‘best hit’ transcript of the other species (column one: *O. scapulalis* transcripts vs *O. nubilalis*) and vice-versa (column two: *O. nubilalis* transcripts vs *O. scapulalis*).

| OHR | *O. scapulalis*  *vs*  *O. nubilalis* | *O. nubilalis*  *vs*  *0. scapulalis* |
| --- | --- | --- |
| [0.0-0.1]  [0.1-0.2]  [0.2-0.3]  [0.3-0.4]  [0.4-0.5]  [0.5-0.6]  [0.6-0.7]  [0.7-0.8]  [0.8-0.9]  [0.9-1.0]  total | 106  147  114  109  106  90  114  92  91  172  1141 | 72  119  101  88  81  94  85  97  87  173  997 |

**Table S5**: **Distribution of Ortholog Hit Ratios (OHR) of PDEGs.**
Table lists the distribution of OHR values of the species-specific transcript (PDEG) aligned with the ‘best hit’ in the NR database.

| GO-ID | Term | Category | FDR | P value | *O. scapulalis* PDEG | *O. nubilalis* PDEG |
| --- | --- | --- | --- | --- | --- | --- |
| GO:0009057 | macromolecule catabolic process | P | 0.8 | 0.000 | 10 | 0 |
| GO:0006508 | proteolysis | P | 0.88 | 0.001 | 9 | 0 |
| GO:0044265 | cellular macromolecule catabolic process | P | 1 | 0.002 | 8 | 0 |
| GO:0019941 | modification-dependent protein catabolic process | P | 1 | 0.004 | 7 | 0 |
| GO:0043632 | modification-dependent macromolecule catabolic process | P | 1 | 0.004 | 7 | 0 |
| GO:0051603 | proteolysis involved in cellular protein catabolic process | P | 1 | 0.004 | 7 | 0 |
| GO:0044257 | cellular protein catabolic process | P | 1 | 0.004 | 7 | 0 |
| GO:0030163 | protein catabolic process | P | 1 | 0.004 | 7 | 0 |
| GO:0048589 | developmental growth | P | 1 | 0.009 | 11 | 2 |
| GO:0048878 | chemical homeostasis | P | 1 | 0.009 | 6 | 0 |
| GO:0016071 | mRNA metabolic process | P | 1 | 0.016 | 11 | 3 |
| GO:0006397 | mRNA processing | P | 1 | 0.016 | 11 | 3 |
| GO:0048638 | regulation of developmental growth | P | 1 | 0.016 | 10 | 2 |
| GO:0050801 | ion homeostasis | P | 1 | 0.021 | 5 | 0 |
| GO:0055080 | cation homeostasis | P | 1 | 0.021 | 5 | 0 |
| GO:0004519 | endonuclease activity | F | 1 | 0.021 | 5 | 0 |
| GO:0009056 | catabolic process | P | 1 | 0.024 | 23 | 12 |
| GO:0000087 | M phase of mitotic cell cycle | P | 1 | 0.027 | 2 | 11 |
| GO:0000279 | M phase | P | 1 | 0.027 | 2 | 11 |
| GO:0008168 | methyltransferase activity | F | 1 | 0.028 | 7 | 1 |
| GO:0061061 | muscle structure development | P | 1 | 0.038 | 11 | 4 |
| GO:0048037 | cofactor binding | F | 1 | 0.038 | 11 | 4 |
| GO:0009888 | tissue development | P | 1 | 0.040 | 17 | 8 |
| GO:0006816 | calcium ion transport | P | 1 | 0.045 | 4 | 0 |
| GO:0006206 | pyrimidine base metabolic process | P | 1 | 0.045 | 4 | 0 |
| GO:0007369 | gastrulation | P | 1 | 0.045 | 4 | 0 |
| GO:0016052 | carbohydrate catabolic process | P | 1 | 0.045 | 4 | 0 |
| GO:0046983 | protein dimerization activity | F | 1 | 0.045 | 4 | 0 |
| GO:0005262 | calcium channel activity | F | 1 | 0.045 | 4 | 0 |
| GO:0004540 | ribonuclease activity | F | 1 | 0.045 | 4 | 0 |
| GO:0000502 | proteasome complex | C | 1 | 0.045 | 4 | 0 |
| GO:0070838 | divalent metal ion transport | P | 1 | 0.045 | 4 | 0 |
| GO:0007610 | behavior | P | 1 | 0.050 | 15 | 7 |

**Table S6: Blast2GO Fisher enrichment test results for PDEGs versus complete transcript set GO terms.**

Single *P* value and FDR (False discovery rate, for multiple testing) are shown by GO term. Category: P for process, F for function, C for compartment. Columns 6 and 7 give the number of Potential Differentially Expressed Gene (PDEG) for the given GO term and for *O. scapulalis* and *O. nubilalis*.

| Identity [%] | OHR | *O. scapulalis* | *O. nubilalis* | Function of the best Insecta NR hits |
| --- | --- | --- | --- | --- |
| 87 | 0.92 | isotig06362 | isotig01010 | A disintegrin and metalloproteinase with thrombospondin motifs 1 (ADAMTS involved in development, blood clotting and turnover of extracellular matrix) |
| 90 | 0.96 | isotig02356 | isotig02085 | Cathepsin L (putative role in insect moulitng) |
| 91 | 0.94 | isotig08012 | isotig07976 | hypothetical protein |
| 93 | 0.93 | isotig00404 | isotig03668 | cuticlin and piopio (role in the properties of the cuticle) |
| 93 | 0.91 | isotig00122 | isotig00198 | myosin III (putative role in vision and photoreception in insects) |
| 93 | 0.91 | isotig06561 | isotig03480 | putative laminin A chain |
| 94 | 0.91 | isotig02811 | isotig05944 | hypothetical protein |
| 94 | 0.95 | isotig10230 | isotig10052 | hypothetical protein |
| 94 | 0.94 | isotig10429 | isotig09367 | DDRGK domain-containing protein 1 |
| 94 | 0.94 | isotig09114 | isotig08426 | hypothetical protein |
| 94 | 0.93 | isotig04888 | isotig04724 | hypothetical protein |
| 95 | 0.93 | isotig09784 | isotig09416 | hypothetical protein |
| 95 | 0.96 | isotig01911 | isotig01050 | brain protein 44 |
| 95 | 0.99 | isotig09996 | isotig03230 | hypothetical protein |
| 95 | 0.96 | isotig11024 | isotig10591 | NADH dehydrogenase 2 |
| 95 | 0.91 | isotig01252 | isotig04895 | gustatory receptor |
| 95 | 0.92 | isotig10853 | isotig10360 | pallbearer (clearance of apoptotic cells) |
| 95 | 0.92 | isotig03013 | isotig07876 | hypothetical protein |
| 95 | 0.97 | isotig07973 | isotig07685 | NADH dehydrogenase (oxidative phosphorylation in mitochondria) |
| 95 | 0.92 | isotig07731 | isotig07571 | hypothetical protein |
| 96 | 0.95 | isotig07918 | isotig07994 | endonuclease-reverse transcriptase |
| 96 | 0.98 | isotig02002 | isotig01736 | hypothetical protein |
| 96 | 0.95 | isotig10692 | isotig10281 | Immediate early response 3-interacting protein 1 (putatively involved in response to pathogenes) |
| 96 | 0.94 | isotig01885 | isotig00388 | ubiquitin-conjugating enzyme E2Q (targets abnormal and short-lived proteins for degradation) |
| 96 | 0.95 | isotig02141 | isotig02117 | hypothetical protein |
| 96 | 0.96 | isotig09592 | isotig09399 | hypothetical protein |
| 96 | 0.94 | isotig05640 | isotig05121 | pleckstrin-like proteiny-like domain |
| 96 | 0.95 | isotig09989 | isotig09246 | hypothetical protein |
| 96 | 0.99 | isotig10256 | isotig09692 | hypothetical protein |
| 96 | 0.99 | isotig09792 | isotig09264 | hypothetical protein |
| 96 | 0.92 | isotig10656 | isotig09708 | hypothetical protein |
| 97 | 0.93 | isotig05427 | isotig05047 | E3 ubiquitin-protein ligase UBR4 |
| 97 | 0.90 | isotig06176 | isotig03311 | hypothetical protein |
| 97 | 0.99 | isotig10467 | isotig10006 | hypothetical protein |
| 97 | 0.93 | isotig08502 | isotig08569 | vacuolar protein sorting protein 18 (segregation of intracellular moleculars into distinct organelles) |
| 97 | 0.91 | isotig06959 | isotig07164 | Hdd1-like protein (regulation of immune system against bacterial infestations) |
| 97 | 0.91 | isotig10833 | isotig10045 | PAK-kinase |
| 97 | 0.91 | isotig00736 | isotig01654 | myophilin (muscle protein, development) |
| 97 | 0.94 | isotig09365 | isotig03289 | phosphatidylinositol 4-kinase beta (involved in cytokinesis) |
| 97 | 0.94 | isotig10733 | isotig10032 | putative GTP-binding protein (putatively involved in olfaction) |
| 97 | 0.97 | isotig09123 | isotig08548 | mitochondrial ribosomal protein S17 |
|  |  |  |  |  |

**Table S7:** **NR-Annotation of homologous transcripts between *O. nubilalis* and *O. scapulalis*, with sequence identities below 97%.**

The table lists the sequence identities (column one), the OHR values (column two), the transcript ids (column three and four) and the common NR annotation, for homologous transcripts (determined by reciprocal best hit) which had sequence identities below 97% between *O. scapulalis* and *O. nubilalis*. Preference was given to annotated functions corresponding to genes of the *Insecta* group.
